# Active Aging and Elderly’s Quality of Life: Comparing the Impact on Literature of Projects Funded by the European Union and USA

**DOI:** 10.2174/1745017901814010001

**Published:** 2018-01-31

**Authors:** I Kirilov, M Atzeni, A Perra, D Moro, MG Carta

**Affiliations:** Department of Medical Sciences and Public Health, University of Cagliari, Cagliari, Italy

## Abstract

**Background:**

The objective of this research is to verify whether European projects on Active Aging (AA) and Elderly Quality of Life (Qol) funded by the Seventh Framework Programme (FP7) produce an impact on literature similar to projects funded by the National Health Institute (NHI) of the United States on international literature using well-known bibliometric indicators. This effort may be useful in developing standardized and replicable procedures.

**Methods:**

Fifteen randomly selected projects on AA and Elderly Qol concluded in August 2017 and funded by FP7 were compared to similar projects funded by the US NHI with reference to papers published (Scopus and Scholar), papers published in Q1 journals, and the number of citations of the papers linked to the projects.

**Results:**

In all the indicators considered, the European projects showed no difference with the US NHI projects.

**Conclusions:**

The EU-funded AA and Qol Elderly projects have an impact on scientific literature comparable to projects funded in the United States by the NHI Agency.

Our results are consistent with the data on general medical research, which indicates that, European research remains at a high level of competitiveness.

In this experimental study, our methodology appeared to be convincing and reliable and it could be applied to the extent of the impact of more extensive research areas.

Our research did not evaluate the relationship between funding required by research and scientific productivity.

## 1 BACKGROUND

Excellence was the one of the declared goals of FP7. Its importance has been reaffirmed with reference to the on-going Horizon 2020 programme [1]. The publications produced in the scientific literature are considered as indicators of excellence by official evaluative documents of the European Commission. As regards specifically FP7 the Annual Monitoring Report 2015 on Horizon 2020 compared the publications reported by FP7 project coordinators to other publications from Member States, the World, Switzerland, United States and Japan [1]. This showed the better bibliometric performance of the publications derived from FP7.

The increases in publications is a crucial point in the light of the European innovation strategy aiming to enable innovative technologies to get faster to the market, and to reinforce the collaboration between industry and academia. This is particularly relevant in the field of psycho-social and healthcare treatments whereby the publication of results of a project is closely related to granting marketing authorization, since psychosocial, psychotherapeutic or pharmacological treatment can pass from experimentation to generalized use only after efficacy tests have been published. Thus, publication of efficacy studies with robust methodologies and convincing results is required for the approbation of a treatment both by European Medicines Agency (EMA) in Europe and the Food and Drug Administration (FDA) in the United States. However, some doubts have been raised about the importance given to the number and the relevance of scientific publications resulted from EU-funded research projects in comparison with other global competitors [2].

With this in mind, it would be useful to develop standardized procedures using well-known bibliometric indicators to assess the impact of projects on the scientific literature.

We attempted to do this in a preliminary study on Active Aging and Quality of Life of Elderly. We chose Active Aging and Quality of Life as research field first because we wanted to focus on a specific and limited area in considering the experimental value of the study; secondly, because this is a research topic of our group, in which we have specific research experience [3-6]; finally, Active Aging is a focal point of research in Europe [7] in relationship to the impressive increase in the percentage of the elderly people in the European Union Members States. In the total population of the Union, elders are estimated to increase from 18.5% (around 95 million elderly persons) in 2014 to 28.7% (around 150 million elderly persons) in 2080 [7].

The objective of this study is to verify, by using well-known bibliometric indicators, whether European projects on Active Aging (AA) and Elderly Quality of Life (Qol) funded by the Seventh Framework Programme(FP7) have an impact on international literature comparable to that of projects funded by the NHI programs in USA, which is the world leader, for the period 1996-2016, in scientific production in the field of Medicine, Public Health, Geriatrics and Psychiatry [8].

This study aims to prepare more comprehensive analysis on the impact of EU-funded research once the on-going Horizon 2020 projects will have been completed.

All the basic current bibliometric indicators are based on citations, that is, on how many times the results of a study are reported in literature: the more a result is considered, the more frequently it is cited. Measuring the impact of a study on the international literature requires a reasonable time for the results to be published and how they become known to the scientific community. Thus, it is not possible, for the time being, to conduct this type of research on the outcome of most projects funded by Horizon 2020, many of which have not yet been completed.

## 2 METHODS

### 2.1 Design

Observational study.

### 2.2 Study Sample

15 projects were randomly selected from the CORDIS database with the following criteria: to be funded by FP7; to be completed by August 2017; to be approved from 1 January 2007 to 31 December 2012. The key words used for the search were “active aging”. With the same frame-time and the same key words 15 projects funded by the US NHI were selected from the NHI database. A block was built for each EU project that included all eligible NHI projects funded ± 1 year with the EU matched project. Then, the 15 US projects were randomly selected, one from each block. The selected projects were automatically excluded if found present in another block.

### 2.3 Variables, Search for Publications and Measure of Citations.

For each project, the number of publications found in the SCOPUS and Google databases were calculated as well as the number of papers published in journals classified as Q1 in one of the fields of the SCIMAGO ranking. For each publication and for each project, the number of citations found was calculated. For the total of the two groups (EU and US) the average number of publications in SCOPUS and Scholar Google databases, the average number of publications in Q1 classified journals, and the average number of citations will be calculated. The citation and the search for papers refer to August 2017.

A search for scientific publications for each selected project in the SCOPUS and Google Scholar databases was carried out. The search was conducted using the name of the project leader, the names of project participants and the title of the project. All project leaders were also contacted by mail with a request to confirm the result of the search and signal any possible missing publications that meet the above-mentioned criteria.

Two researchers in blind curried out the search for publications and the verification of the affiliation of a publication to a project, in case of disagreement a third researcher was called into question as an arbitrator. The affiliation of a publication to a project was easily for US project because the citation of the project is mandatory for the publications funded by; was quite more problematic in UE.

### 2.4 Data Analysis

The variables on numeric scales will be treated with the ANOVA one-way analysis of variance, the variables on a nominal basis with the chi square test.

### 2.5 Ethics

The study was carried out in agreement with the ethical principles of the Helsinki Declaration. The ethics committee of the Azienda Mista Ospedaliero Universitaria di Cagliari approves the study. All data treated in this study were already published and of public access.

## 3 RESULTS

Table **1** summarizes the results of the research. For all the indicators considered (number of published papers indexed in Scopus; number of papers published in scientific journals classified in the first quartile (Q1) of the Scimago ranking; mean number of citations in journals indexed in the Scopus depository; mean number of citations in journals indexed in the Google Scholar depository), no difference between the European projects and the US NHI projects has been found. The projects with no citation of their publications in the journals indexed in the Scopus depository were 5 (33.3%) in the European sample and 2 (13.3%) in the American sample; this difference did not reach statistical significance (χ2 with Yates correction = 0.83; p=0.36). The projects with no citation of their publications in the journals indexed in the Google depository were 4 (26.6%) in the European sample and 2 (13.3%) in the American sample; this difference did not reach statistical significance (χ2 with Yates correction = 0.75; p=0.38).

## 4 DISCUSSION

The study has demonstrated that the results of EU-funded Active Aging projects have an impact on international scientific literature and a level of visibility comparable to that of similar projects funded in the United States by the NHI Agency.

We consider that the choice of the two depositories for the calculation of citations (Scholar and Scopus) has not produced any bias. This choice was motivated by the fact that one of them is European (Scopus), while the other one is American. The Web of Science database is also widely used in literature, but 99% of Web of Science indexed papers are also in Scopus and virtually 100% are indexed in Scholar.

The results of this study must however be considered in the light of some methodological limits. The first is the low statistical power of the study owing to the small size of the sample examined. This requires the data to be confirmed by more extensive studies, which we plan to carry out once most of the European projects funded by Horizon 2020 have been completed. Thanks to the more extensive study, we will be able to confirm or refuse the tendency towards better performance in the American sub-sample as regards almost all the indictors considered, which however in the current study does not reach statistical significance.

The second refers to the fact that in the publications derived from NHI projects it is mandatory to report the precise indication of the financing program and the official number of approval. This makes it impossible to miss a publication financed by a single project. In addition, researchers are highly motivated, and to certain extent oblige to publish, the results of their work funded by the NHI programs, in a way that if they fail to do so, this may reduce significantly the chances to receive NHI finance in the future. Moreover, we have found many European publications clearly resulting from projects funded by FP7 in which the source of funding was not mentioned. Thus, the different perception and rules regarding the scientific publications in Europe and in USA may have led to an underestimation of European publications.

The different accent put on the need to publish results and be visible for the international scientific community is also linked to the greater importance attributed by the US and to science and research, and to the stronger link between research and innovation.

Although the considerable effort made in the recent years, in particular regarding the EU budget devoted to research and innovation, the Union, as a whole, still lags behind its main international competitors. Concerning domestic spending on research measured as a percentage of GDP of the total expenditure on research by all resident companies, both public and private in a given country, we see that the European Union increased from 1.70 in 1996 to 2.04 in 2013 according to the World Data Bank [9] and from 1.67 in 2000 to 1.96 in 2015 according to OECD [10].In contrast, in the US it was 2.44% in 1996 (data for 2013 are unavailable in the World Data Bank [9]) and according to OECD it increased expenditure for research from2.61% in 2000 to 2.69% in 2015 [10],while China increased from 0.57 in 1996 to 2.05 in 2013 according to the World Data Bank [9] and from 0.83 in 2000 to 2.07 in 2015 according to OECD [10]. Thus, China has also surpassed Europe as a percentage of funds for research.

In addition, investment in research and innovation are far from being homogeneous within the Union. In some countries, even among those with large number of population, such as Italy, they are surprisingly scanty, and this lowers the overall European average. Although the analysis of competitiveness indicates that surprisingly Italy maintains an acceptable level of competitiveness in research despite its low level of investments [11].

Our results on a relatively small but very impactful scientific field are consistent with data on general medical research, which indicates that European research remains at a very high level of competitiveness. According to SCIMAGO ranking, even considering the specific countries, the US is the main producer of scientific literature in the medical field; but the total number of scientific papers produced in journals indexed in the period from 1996 to 2015 summing the 27 EU states (excluding the UK) in medical sciences is 3,510,689 against 3,227,211 of the US, 930,273 of the UK, 673,105 of Japan, and 594,791 of China (Scimago 2017). It should thus be noted that in a well-supported sector such as active aging, European research was found to have an impact on literature as compared to the impact of US projects in the same field.

In this observational evaluative study, our methodology appears to be reliable and could be applied to assess the impact of more extensive research areas. However, a correct evaluative methodology focused on published papers can only be established if it becomes mandatory in Europe for publications to report the essential identification of the funding projects. This would also encourage authors to gain greater international visibility, with clear positive impacts on the Union's image.

The cost effectiveness of the projects examined, that is, the relationship between the funding for research and the scientific productivity, is out of the scope of this study, but will be analyzed in a later stage.

## CONCLUSION

The EU-funded on AA and Qol Elderly projects have an impact on scientific literature comparable to projects funded in the United States by the NHI Agency.

Our results are consistent with data on general medical research, which indicates that, European research remains at a high level of competitiveness.

In this experimental study, our methodology appeared to be convincing and reliable and it could be applied to the extent of the impact of more extensive research areas.

Our research did not evaluate the relationship between funding required by research and scientific productivity

## Figures and Tables

**Table 1:** Synthesis of the results

NIH DATA BANK					HORIZON DATA BANK			
	Total of Publications	Papers in Q1	N. Citations on Scopus	N. Citations on Scholar	Total of Publications	Papers in Q1	N. Citations on Scopus	N. Citations on Scholar
I PROJET	2	1	0	79	6	3	198	390
II PROJET	13	10	22	349	2	1	0	0
III PROJET	9	8	18	358	7	1	34	57
IV PROJET	14	8	81	196	42	25	689	1108
V PROJET	14	13	347	684	16	6	34	77
VI PROJET	13	10	480	675	0	0	0	0
VII PROJET	3	3	8	103	0	0	0	0
VIII PROJET	4	1	14	101	1	1	380	555
IX PROJET	4	2	72	97	11	9	185	283
X PROGETTO	0	0	0	0	0	0	0	0
XI PROJET	1	1	21	42	10	1	43	92
XII PROJET	13	10	165	309	4	1	13	22
XIII PROJET	4	3	56	93	3	2	95	158
XIV PROJET	0	0	0	0	0	0	0	0
XV PROJET	12	10	92	117	12	3	116	219
	7.07 +/- 5.59	5.3 +/-4.6	91.00+/-140.81	213.5+/- 221.0	5.2 +/-5.3	3.5 +/-6.4	119.13+/-190.20	207.42+/-310.5
STAT	df 1,28,29 F=0.88 P=0.35	df 1,28,29 F=0.78 P=0.38	Df 1,28,29 F=**0.213** **P=0.648**	df= 1.28.29 F=0.004 P=0.95	–	–	–	–
